# Supplementary material for: Plasma Membrane Ca2+ ATPase Isoform 4 (PMCA4) Has an Important Role in Numerous Hallmarks of Pancreatic Cancer
Source: Cancers (Basel). 2020 Jan 16;12(1):218. doi: 10.3390/cancers12010218 (PMC7016988; doi:10.3390/cancers12010218)

*Supplementary material*

Plasma membrane Ca^2+^ ATPase isoform 4 (PMCA4) has an important role in numerous hallmarks of pancreatic cancer.

Pishyaporn Sritangos, Eduardo Pena Alarcon, Andrew D. James, Ahlam Sultan, Daniel A. Richardson, Jason I.E. Bruce


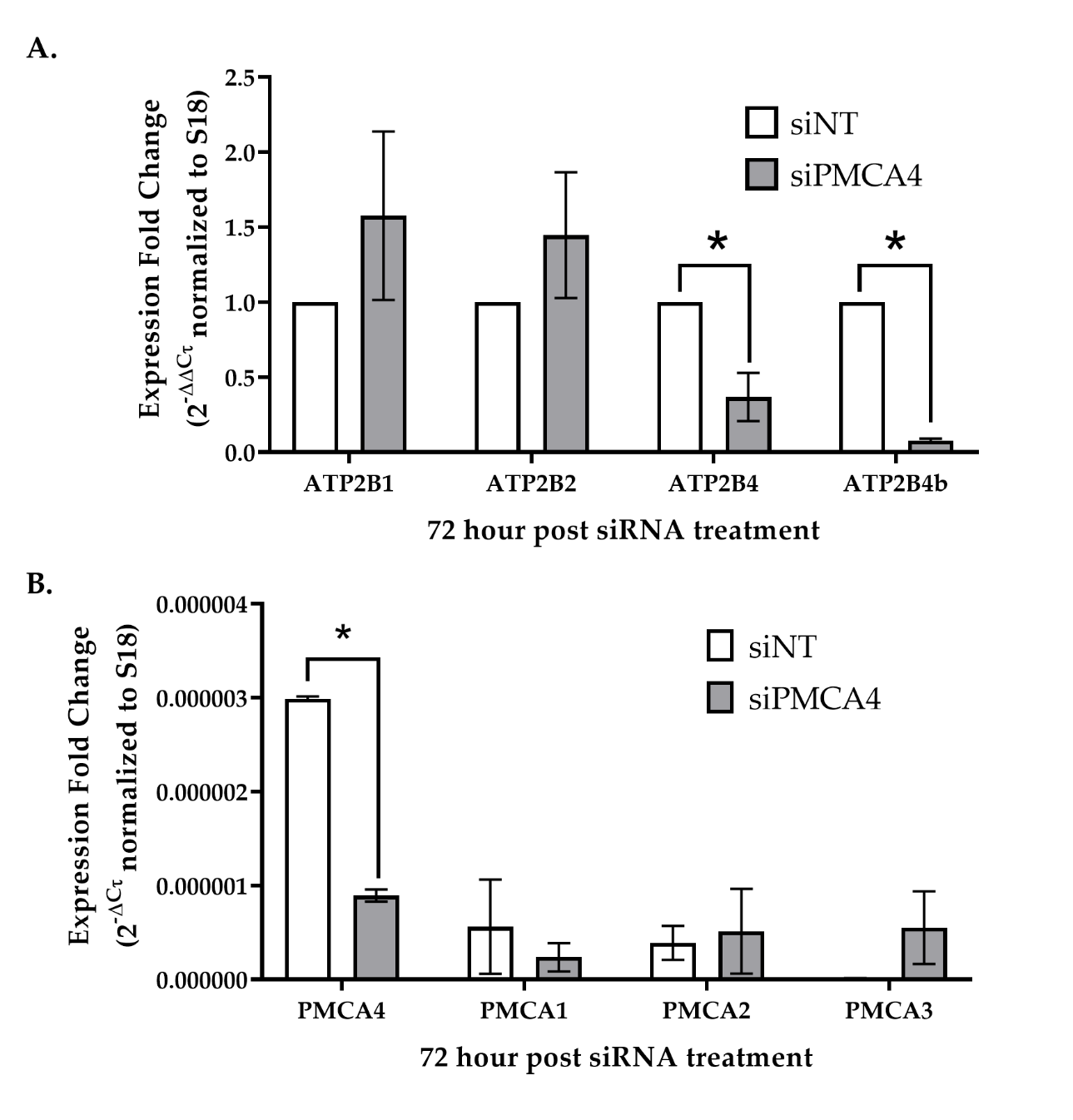


**Figure S1.** PMCA4 knockdown does not lead to significant upregulation of other PMCA isoforms. MIAPaCa-2 cells seeded with either 25 nM siNT control or siPMCA4 for 72 hours. mRNA expressions of PMCA1-4 were examined using RT-qPCR and were normalized to S18 rRNA housekeeping gene. **(A)** The relative expression of siPMCA4 was compared to siNT. Data are presented as relative mRNA expression with respect to siNT (2^-ΔΔCτ^). **(B)** The relative expressions of PMCA1-4 mRNA were normalized to S18 rRNA. Statistical significance was determined using Mann-Whitney non-parametric t-test. * represents a statistically significant difference between siNT and siPMCA4, where P<0.05. (N=3, 4 replicates per treatment condition).

**
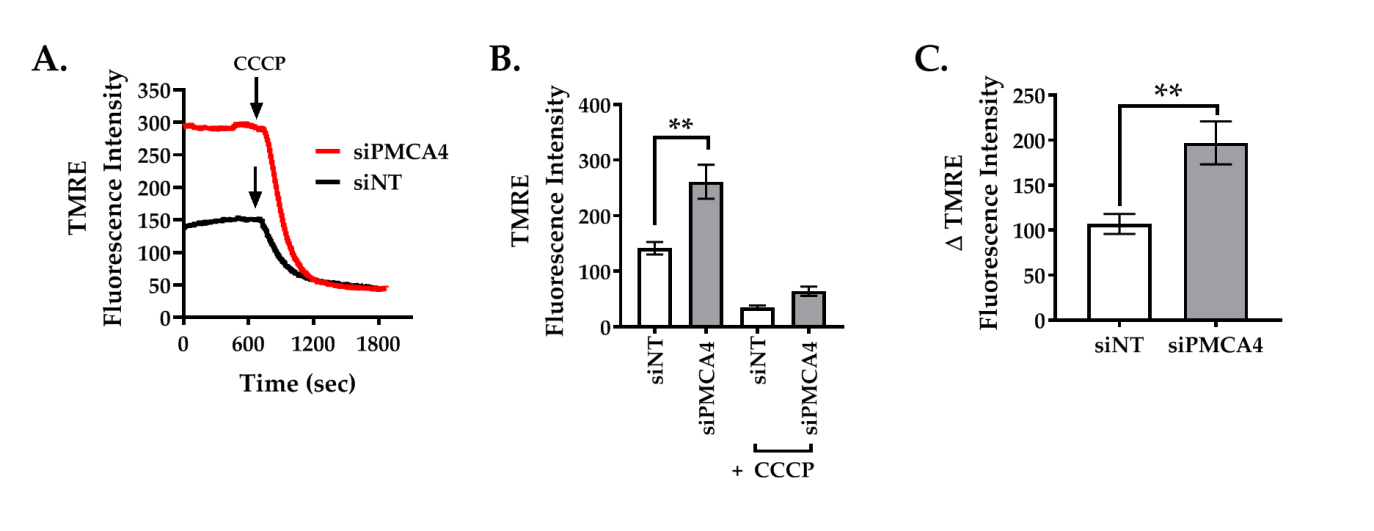
**

**Figure S2.** PMCA4 knockdown increased basal mitochondrial membrane potential. MIAPaCa-2 cells seeded with either 25 nM siNT control or siPMCA4 for 48 hours. **(A)** Representative TMRE traces are shown with arrows indicating the addition of CCCP. **(B)** Raw TMRE fluorescence intensity of the basal and post-CCCP addition **(C)** Mitochondrial membrane potential is shown as a change in TMRE fluorescence intensity. ** represents a statistically significant difference between siNT and siPMCA4, where P<0.005. (N≥4, at least 50 independent cells were analysed per treatment condition).


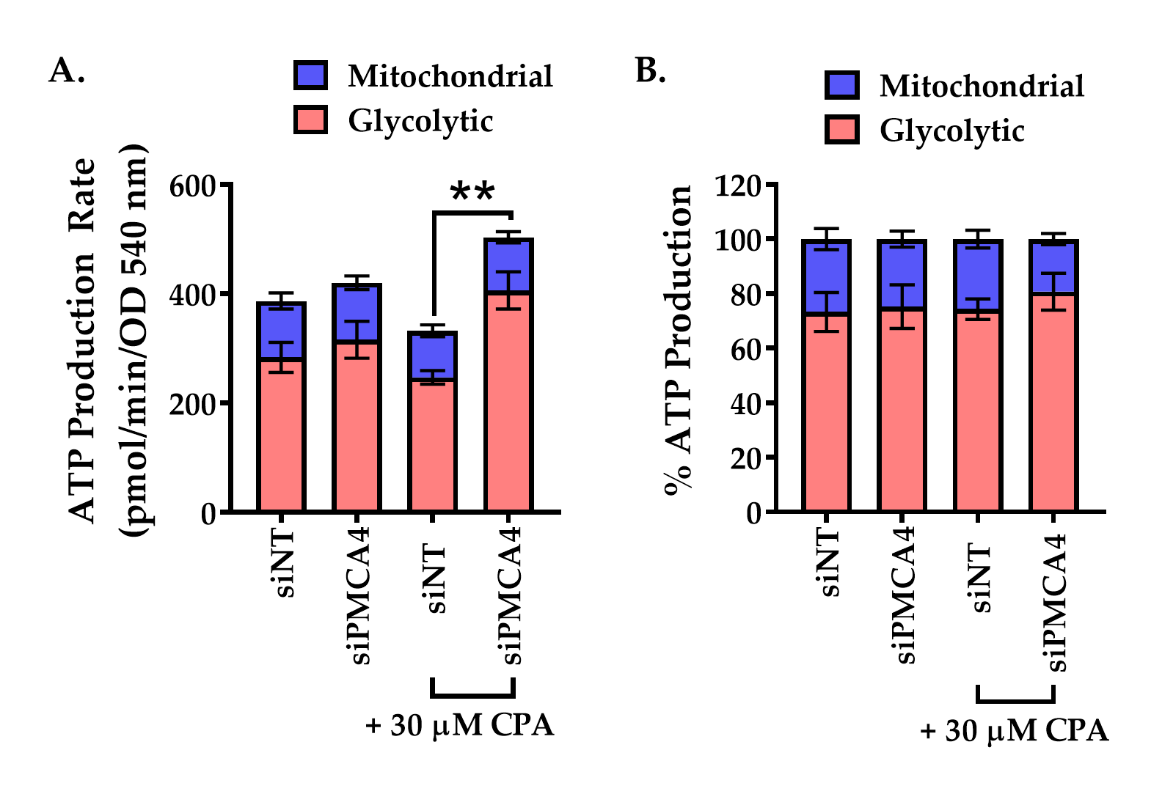


**Figure S3**. Effect of PMCA4 knockdown on ATP production rate. MIAPaCa-2 cells seeded with either 25 nM siNT control or siPMCA4 for 48 hours. Ca^2+^ stress was induced by 30 μM cyclopiazonic acid (CPA). Agilent Seahorse ATP Production Rates were determined from changes in oxygen consumption rate (OCR, pmol/min) and ECAR (mpH/min). **(A)** The raw ATP production rates are present as pmol/min normalized to protein concentration (OD 540 nm). **(B)** The ratios of mitochondrial vs glycolytic ATP production are presented as % of ATP production, using a stacking bar graph. Statistical significance was determined using the Kruskal-Wallis test. ** represents a statistically significant difference between CPA-treated siNT and siPMCA4, where P<0.01. (N=3, 4 replicates per treatment condition).


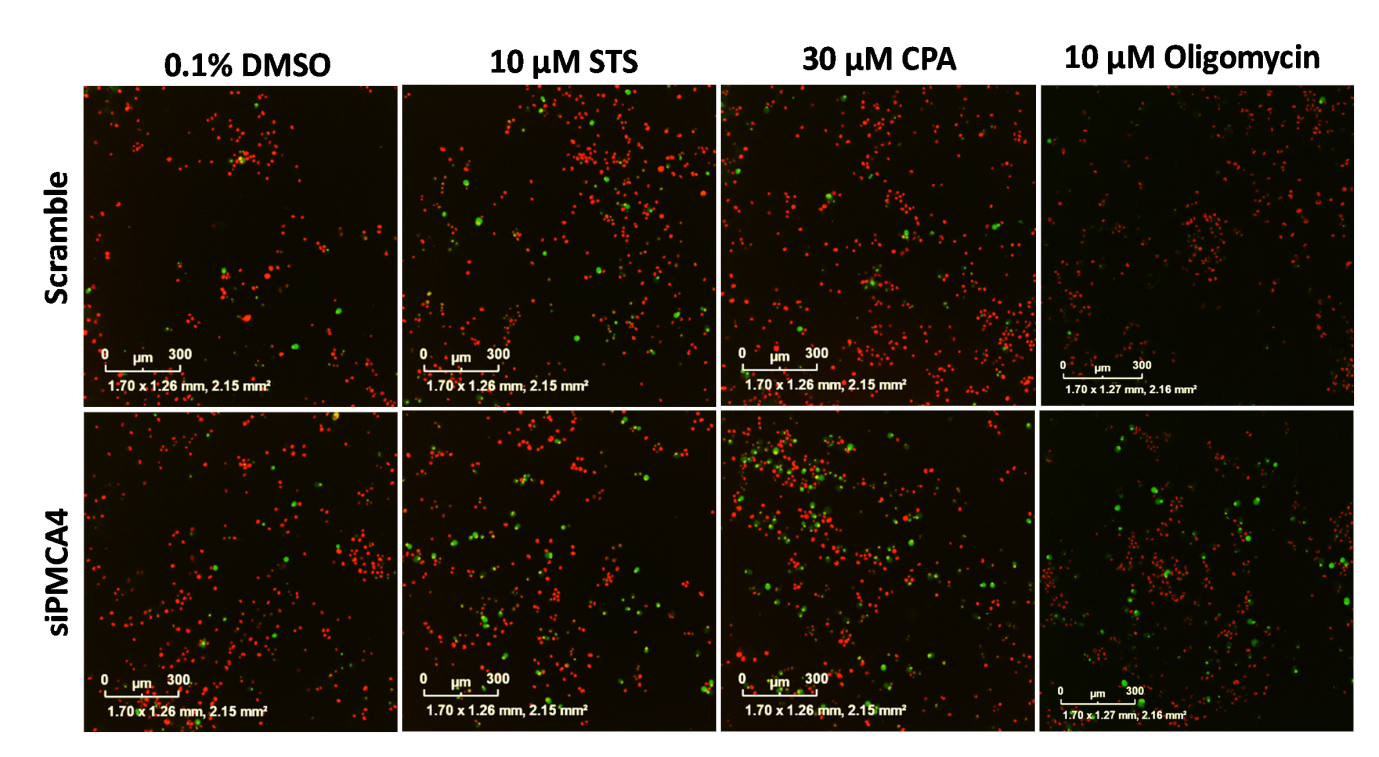


**siPMCA4**

**siNT**

**Figure S4.** PMCA4 knockdown sensitizes MIA-PaCa-2 PDAC cells to apoptotic cell death. Representative images of non-targeting (siNT) versus PMCA4 knockdown MIAPaCa-2 cells treated with either 0.1% DMSO vehicle control (VC), apoptotic inducing staurosporin (STS), cyclopiazonic acid (CPA), oligomycin (OM) or iodoacetate (IAA). The cells are labelled with Nuclear ID red (red) and Caspase3/7 reagent (green). Cells were then imaged using the Incucyte, on the red (excitation 565-605 nm; emission 625-705 nm) and green channel (excitation 440-480 nm; emission 504-544 nm), using a 10x objective lens. (N=3-4, 3 replicates were performed per treatment condition).

**Method S1.** **Data mining search filter –** Oncomine public database data mining was performed using [www.oncomine.org](http://www.oncomine.org). The specific search term filters included: “ATP2B4 (gene)”, “Analysis Type: Cancer vs. Normal Analysis”, and “Cancer Type: Pancreatic Cancer”. This search should yield multiple studies (including Badea Pancreas) which compared the ATP2B4 expression in Cancer vs. healthy tissues data. Alternatively, an existing Oncomine user may follow the following link: https://www.oncomine.org/resource/main.html#d%3A149671257%3Bdso%3AgeneOverex%3Bdt%3Adataset%3Bec%3A%5B2%5D%3Bepv%3A150001.151078%2C2937%2C3508%3Bet%3Aover%3Bf%3A1900610%3Bg%3A493%3Bp%3A149672127%3Bpg%3A1%3Bpvf%3A3471%2C5448%2C150004%3Bscr%3Adatasets%3Bss%3Aall%3Bv%3A18

Similarly, the Human Protein Atlas database search was performed using [www.proteinatlas.org](http://www.proteinatlas.org). The specific search term is “ATP2B4”. The Cancer Genome Atlas (TCGA) PDAC patient survival data were then obtained by selecting the “Pathology” tab then “pancreatic cancer”, available under “RNA expression overview”. Alternatively, follow the following URL: <https://www.proteinatlas.org/ENSG00000058668-ATP2B4/pathology/pancreatic+cancer>. All survival data used in this study were obtained from “TCGA RNA samples” data section available on the website.

**Method S2. Ca^2+^ clearance assay –** PMCA activity was measured by *in situ* Ca^2+^ clearance assay [1]. MIAPaCa-2 cells were perfused with HPSS in the absence of external Ca^2+^ with 1 mM of EGTA, a divalent cation chelator [2], and 30 μM of cyclopiazonic acid (CPA). CPA was used to inhibit sarcoendoplasmic reticulum calcium ATPase (SERCA) and prevent Ca^2+^ uptake into the ER stores [3], enabling passive Ca^2+^ leak from the ER. This induced a transient increase in cytosolic [Ca^2+^]_i_ and PMCA activity was assumed to restore the baseline [Ca^2+^]_i_ [4]. Subsequent perfusion with 20 mM Ca^2+^ HPSS supplemented with CPA (20 mM Ca^2+^ HPSS) induced a rapid [Ca^2+^]_i_ increase due to store-operated Ca^2+^ entry (SOCE). After the Ca^2+^ influx phase reached a maximum plateau, external Ca^2+^ was again removed (1 mM EGTA and CPA HPSS buffer without Ca^2+^; 0 Ca^2+^ HPSS) to induce Ca^2+^ clearance [44]. In Ca^2+^ clearance experiment (e.g. siRNA knockdown), where only a single clearance curve is generated, a single exponential decay fit was often used for Ca^2+^ clearance analysis where the average time constant were compared between the treatment and controlled condition. The Ca^2+^ clearance rate was measured by fitting the clearance phase to a single exponential decay to yield the time constant (tau, τ) [1,4,5].


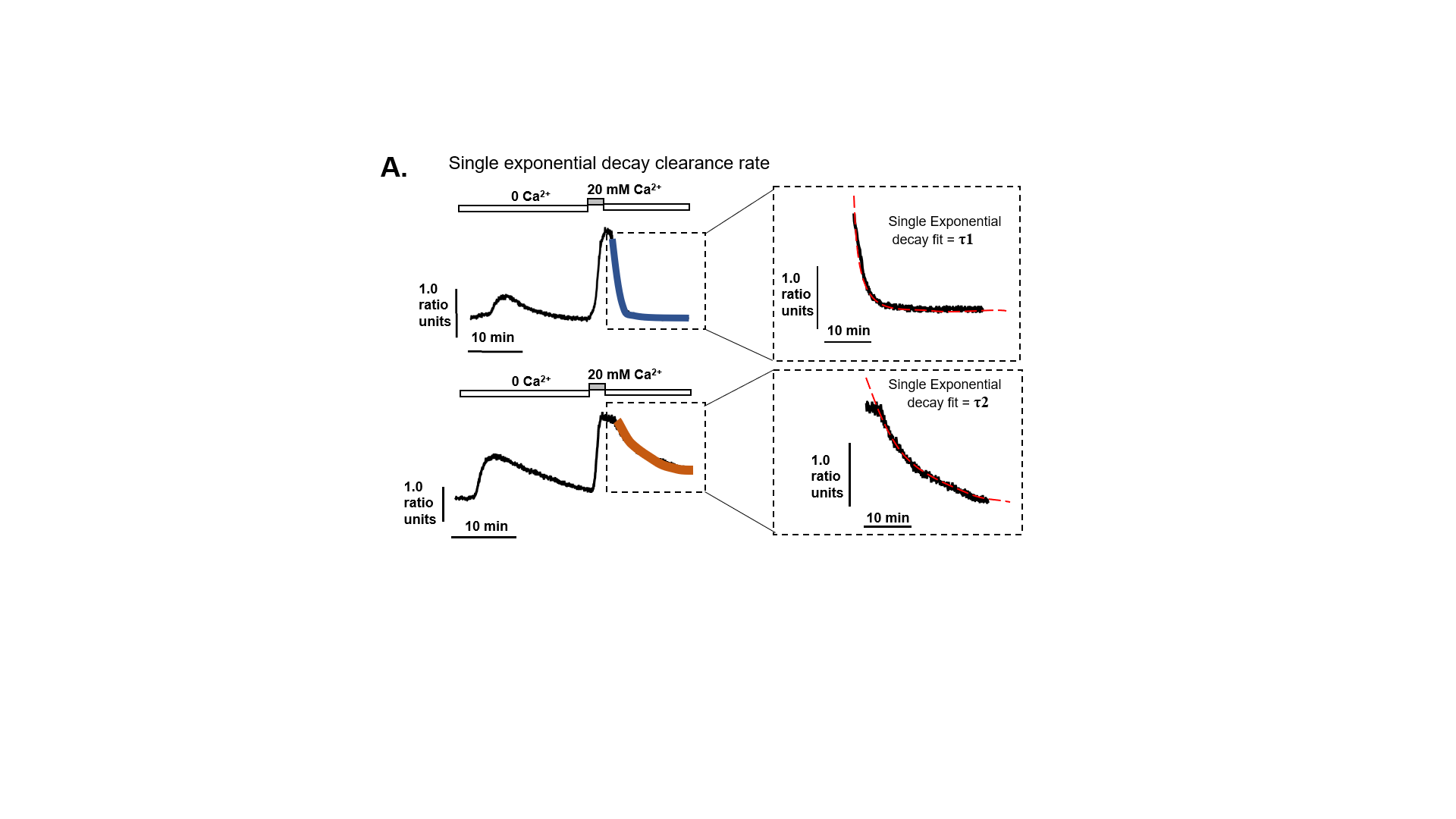


**Figure S5.** Ca^2+^ clearance rate calculation methods. **(A)** The single exponential decay clearance rate was performed on siRNA treated cells which displayed less dramatic inhibition of PMCA activity. As siRNA knockdown process requires a period of 48-72 hr, a paired experimental design cannot be achieved. The clearance phase of siRNA treated cells was obtained and fitted to a single exponential decay curve. The τ obtained represents the clearance rate. White bar represents 0 Ca^2+^ HPSS containing 30 µM CPA while the grey bar represents 20 mM Ca^2+^ HPSS containing 30 µM CPA. The blue line represents clearance phase 1 (control) and the orange line represents clearance phase 2 (treated). The expanded boxes shows the single exponential decay fit (red dash line) to individual clearance phase, yielding clearance rate τ1 (control) and clearance rate τ2 (treated), respectively.

**Method S3.** **Fura-2 Ca^2+^ concentration calibration –** As fura-2 340/380 nm fluorescence ratio represents the Ca^2+^ bound dye/Ca^2+^ free dye status, this relative fura-2 ratios ratio is proportional to [Ca^2+^]. The equation established by Grynkiewicz *et al* (1985) could be used to calculated [Ca^2+^] from fura-2 ratio [6] as follows:

$$\left[ \boldsymbol{Ca}^{\boldsymbol{2+}} \right]_{\boldsymbol{i}}\boldsymbol{=}\boldsymbol{K}_{\boldsymbol{d}}\boldsymbol{\times}\left( \frac{\boldsymbol{R -}\boldsymbol{R}_{\boldsymbol{min}}}{\boldsymbol{R}_{\boldsymbol{max}}\boldsymbol{- R}} \right)\boldsymbol{\times}\left( \frac{\boldsymbol{Sf}_{\boldsymbol{380}}}{\boldsymbol{Sb}_{\boldsymbol{380}}} \right)$$

Following this equation, [Ca^2+^]_i_ represents intracellular Ca^2+^ concentration and K_d_ represents the cytosolic Ca^2+^ binding affinity of fura-2 (225 nM, as determined by Grynkiewicz *et al* (1985)). In order to translate this 340/380 nm fluorescence ratio (R) into meaningful intracellular Ca^2+^ concentrations ([Ca^2+^]_i_), the minimum ratio (R_min_) and maximum ratio (R_max_) as well as the raw fluorescence 380 nm signal of the fura-2 in the absence (Sf_380_; Ca^2+^ free dye state) and the presence of Ca^2+^ (Sb_380_; Ca^2+^ bound dye state) must be determined.

Using 4 μM fura-2 loaded MIAPaCa-2 cells, these calibrations were performed by firstly by perfusion with Ca^2+^ free HPSS (supplemented with 1 mM EGTA). Once a stable basal fura-2 ratio has been established, the perfusion was stopped and 10 μM ionomycin (#BP25271, Fisher Scientific) was added to the static bath for 2 minutes. As an ionophore, ionomycin was employed to facilitate the depletion of intracellular Ca^2+^ stores [7]. Afterwards, further perfusion with Ca^2+^ free HPSS with 1 mM EGTA was continued to facilitate cellular Ca^2+^ removal until a stable R_min_ was reached. Later perfusion with 20 mM Ca^2+^ HPSS was done to yield the R_max_. Using Grynkiewicz *et al* (1985) equation, the [Ca^2+^]_i_ of each individual cells could be calculated, converted into log[Ca^2+^]_i_ and plotted against fura-2 ratio to establish a concentration curve (Figure S5). Through multiple independent calibrations experiments, a robust calibration curve was generated from 30-50 individual MIAPaCa-2 cells, enabling the extrapolation of this calibration for other fura-2 experiments done in MIAPaCa-2 cells. It should be noted, however, that fura-2 detection limit is reported to be 1 nM [8] and its saturation is reported to occur at 1 μM Ca^2+^ [9]. Therefore, any calculated [Ca^2+^]_i_ value that fell out of this narrow 1-1000 nM range may not be an accurate estimation. Thereby, siPMCA4 Ca^2+^overload data could not be fitted to the Ca^2+^ calibration as the [Ca^2+^]_i_ measured exceeded the accurate detection range of 1 μM.


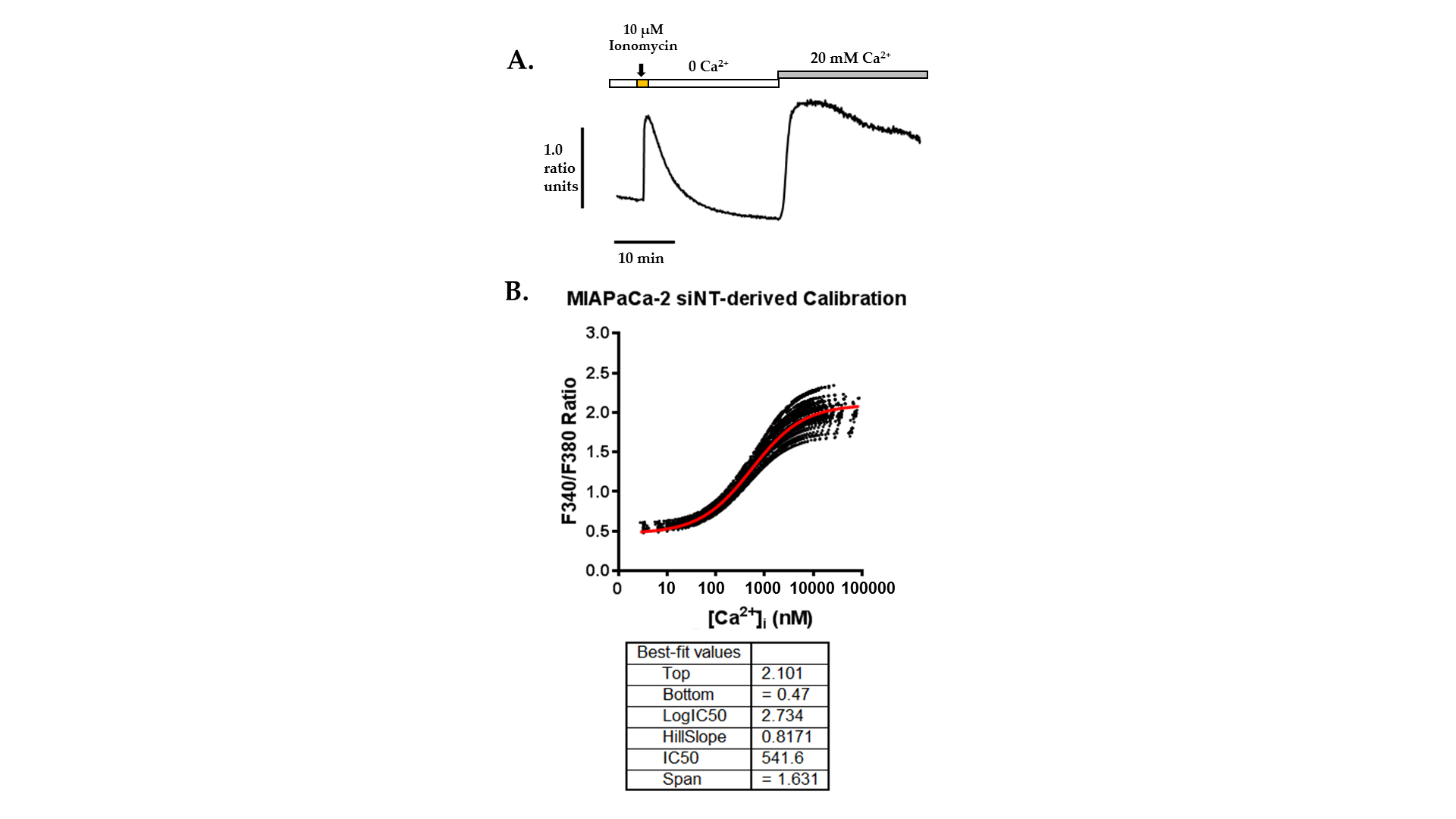


**Figure S6.** Fura-2 Ca^2+^ calibration curve. **(A)** Representative trace of Ca^2+^ calibration curve. White bar represents 0 Ca^2+^ HPSS perfusion whereas the gray bar represents 20 mM Ca^2+^ HPSS perfusion. The purple bar represents the treatment of 10 µM ionomycin under static bath condition. **(B)** Ca^2+^ calibration curved obtained from 30 individual siNT treated (48-72 h) MIAPaCa-2 cells. Black dotted lines represent data derived from individual cells. Red line represents a sigmoidal best-fit data. The key values in the table represent the averaged best-fit data: Top: R_max_; Bottom: R_min_; IC_50_: Kd.

**Method S4.** **Gap size analysis using Fiji ImageJ software.** Cell-free gap images were collected on an Olympus IX83 inverted microscope using a 4x / 0.13 UPlanFL N objective and captured using an Orca ER camera (Hamamatsu, Japan) through CellSens software (Olympus, Tokyo, Japan). Cell-free gap areas were then analysed using Fiji ImageJ software (Fiji ImageJ (http://imagej.net/Fiji/Downloads). Gap images were imported into Fiji ImageJ using the menu “Plugin > Bio-Format > Bio-Format Importer”. Spatial calibration of the captured gap area was done based on the Orca camera pixel size. For instance, images captured using a 4x lens and 1x1 binning, the pixel size is determined to be 1.6125 µm/1 pixel (The University of Manchester Bioimaging Facility). Thereby, using the Fiji ImageJ “Analyze > Set Scale” menu, a scale can be set based on the known pixel size to measure the cell-free gap area. Calibrated images were transformed into binary images to clearly distinguish the cell-free area. The gap areas were then identified using the “Analyze > Analyze Particle” menu. Gap areas measured were then exported into an excel spread sheet for further analysis.

**Method S5.** **Analysis of ATP production rate from existing Mito Stress Test data.** The recent availability of the Agilent Seahorse XF Real-Time ATP Rate Assay Kit has provided a new means to monitor the dynamic ATP production rates in live cell. Using two sequential injections of 1.5 μM OM and 0.5 μM Rot/AA, the assay relies on the changes in OCR and ECAR which could be used to calculate total ATP production rate, mitochondria ATP (mitoATP) production rate and glycolysis ATP (glycoATP) production rate [10] as follows:

| **Parameter** | **Parameter Equations** |
| --- | --- |
| mitoATP  Production Rate | [(Last OCR rate measurement before first injection - Minimum OCR rate measurement after OM but before Rot/AA injection) x 2 x (P/O)] |
| glycoATP  Production Rate | Last glycoPER measurement before the first injection |
| Total ATP  Production Rate | (mitoATP Production Rate) + (glycoATP Production Rate) |
| XF ATP Rate Index | (mitoATP Production Rate) / (glycoATP Production Rate) |

**Table S1.** Parameters equations of Agilent Seahorse XF real-time ATP rate assay. Abbreviations: ATP: adenosine triphosphate; mitoATP: mitochondrial respiration derived ATP; glycoATP: glycolysis derived ATP; OCR: oxygen consumption rate; ECAR: extracellular acidification rate; glycoPER: glycolysis derived proton efflux rate; P/O: theoretical number of ATP synthesized per oxygen atom; OM: oligomycin; Rot/AA: rotenone/antimycin A mixture. Table modified from the manufacturer’s user guide [11].

As these parameters, required for ATP rate calculations, could also be obtained from Mito stress test data, we deemed that it was possible to calculate ATP production rate from the glycoPER readings of existing Mito stress results. The changes in OCR in combination with the theoretical ratio of number of ADP phosphorylated to ATP per oxygen atom (P/O, suggested to be 2.75 [10]), enables the calculation of mitoATP production rate. On the other hand, the calculation of glycoATP production rate is obtained from the ECAR readings which are converted into proton efflux rate (PER) based on the Agilent Seahorse media buffer factor used (i.e. Buffer factor = 2.4 mmol/L/pH for Seahorse XF DMEM, pH 7.4) and the CO2 contribution factor (CCF = 0.61 ± 0.13 for XFe96 and XF96 Analyzer [12]). Rot/AA mediated inhibition of complex I and III lead to complete inhibition of mitochondrial respiration, enabling the identification of mitochondrial-associated acidification.


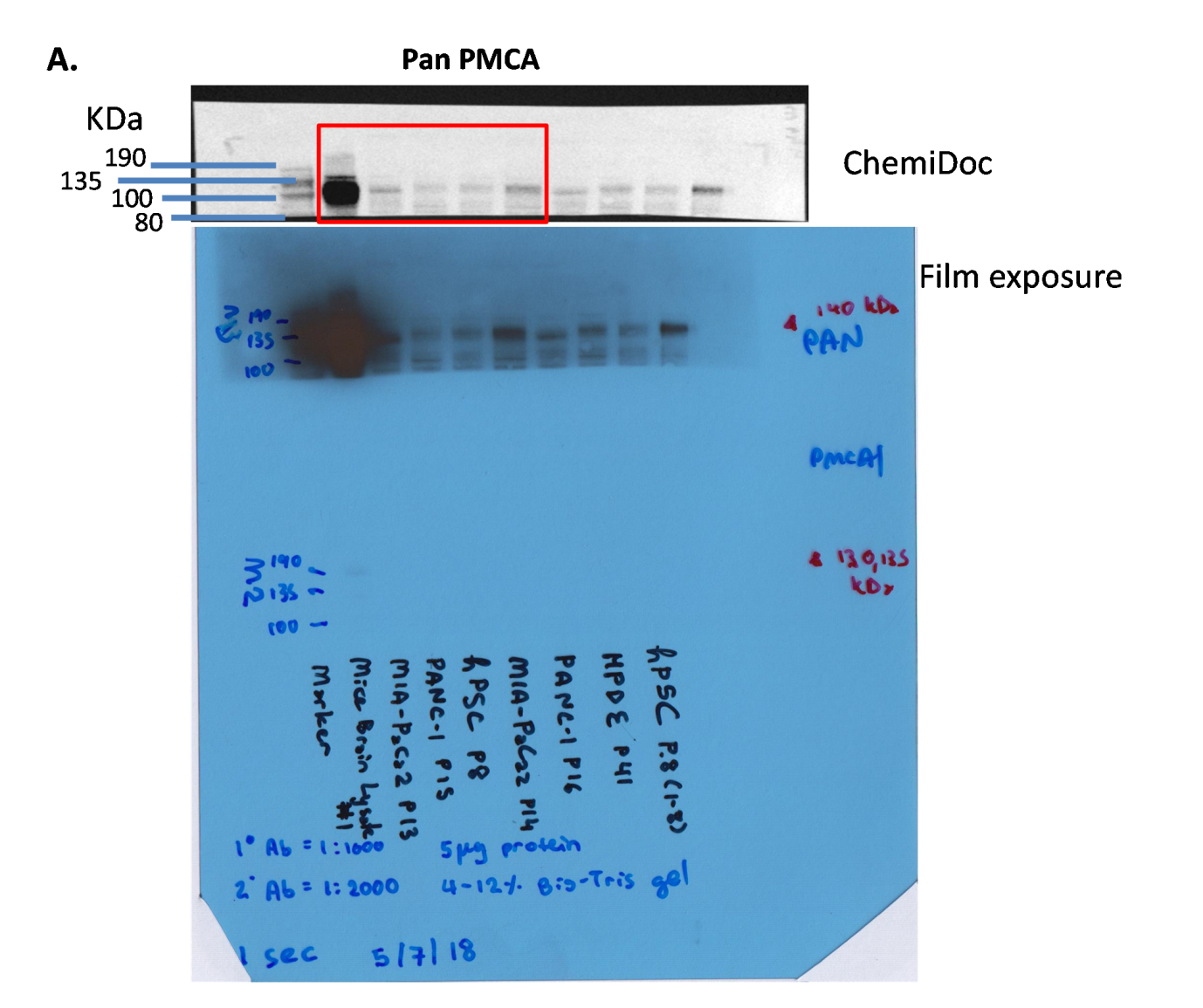


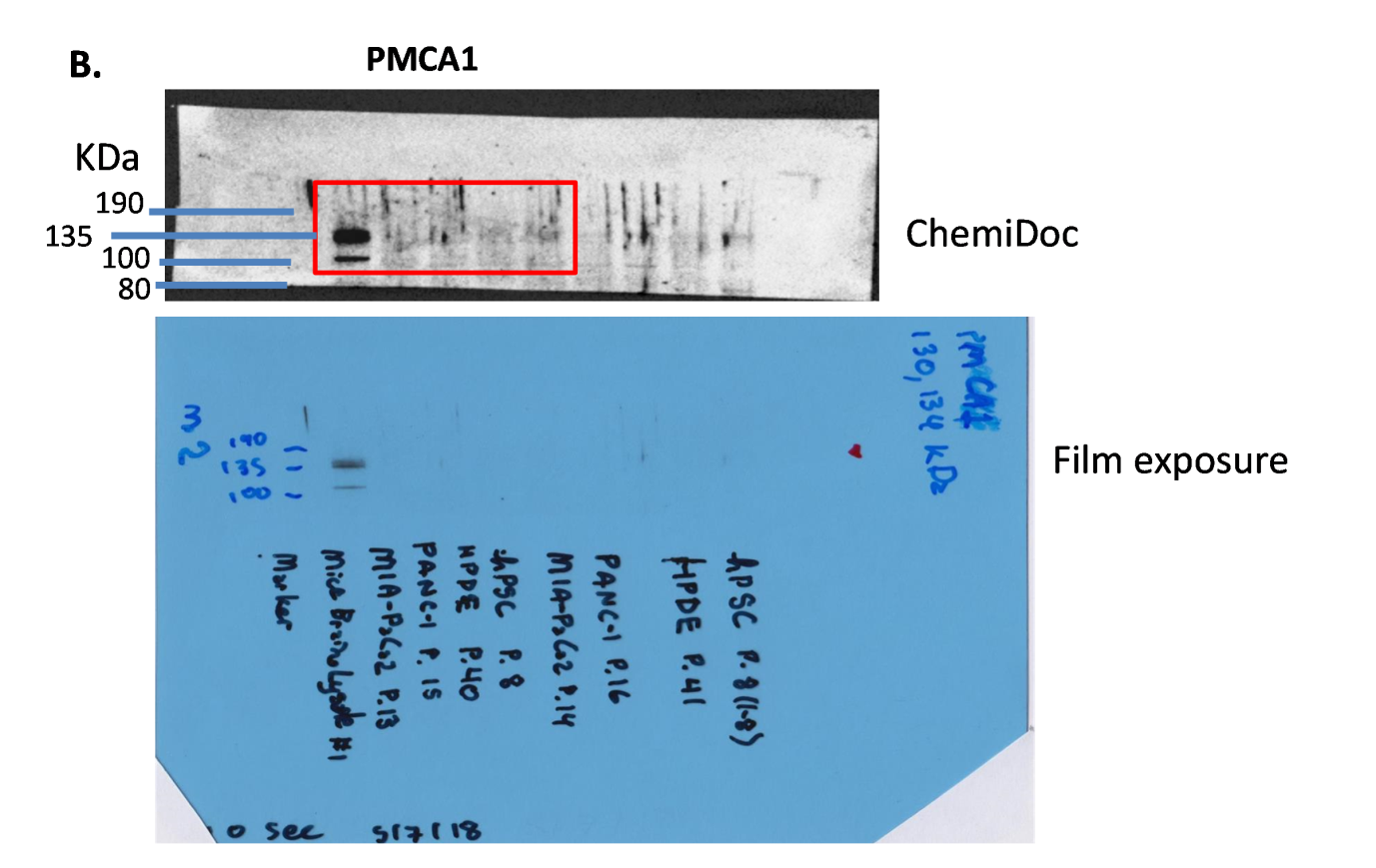


**Figure S7A-B.** Continuing next page


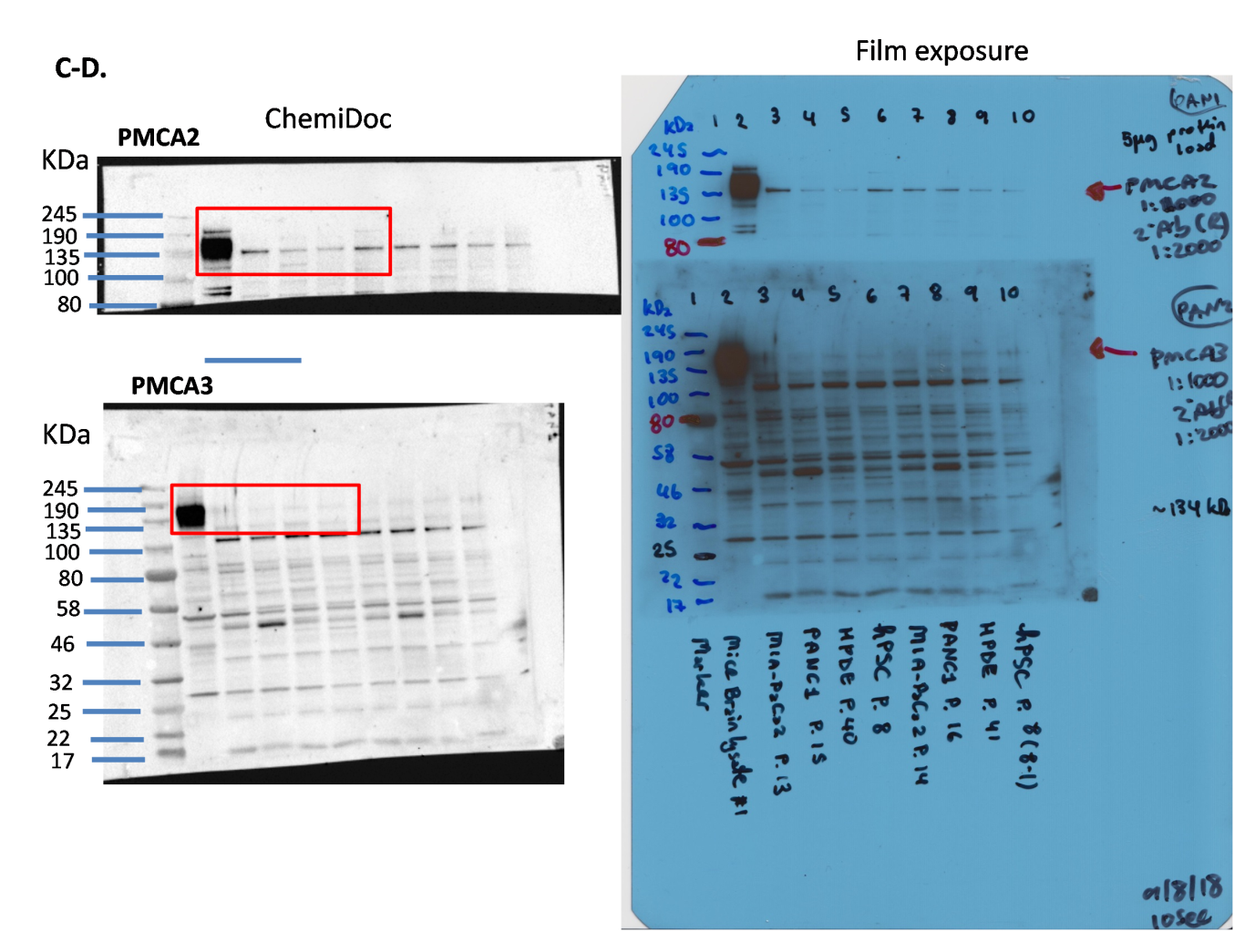

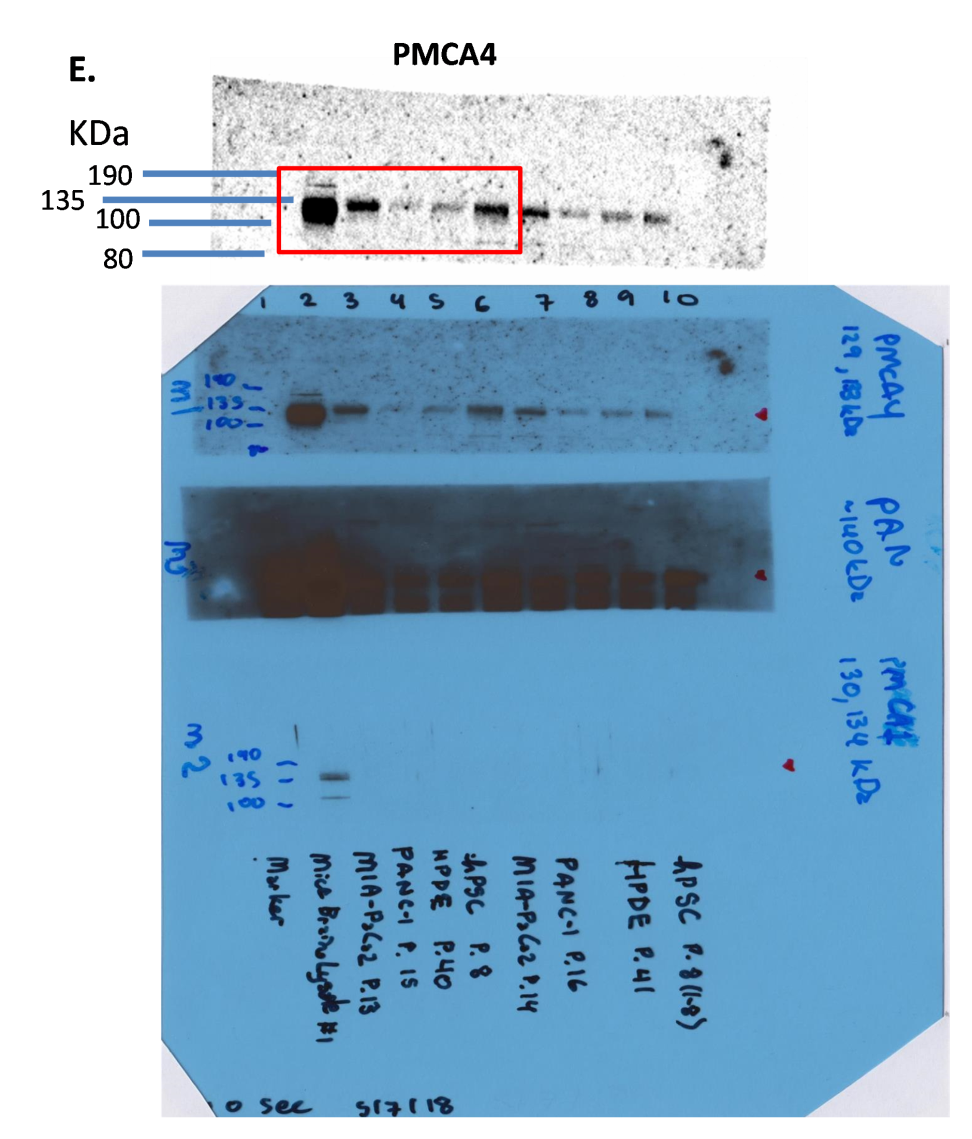


**Figure S7C-E.** Continuing next page


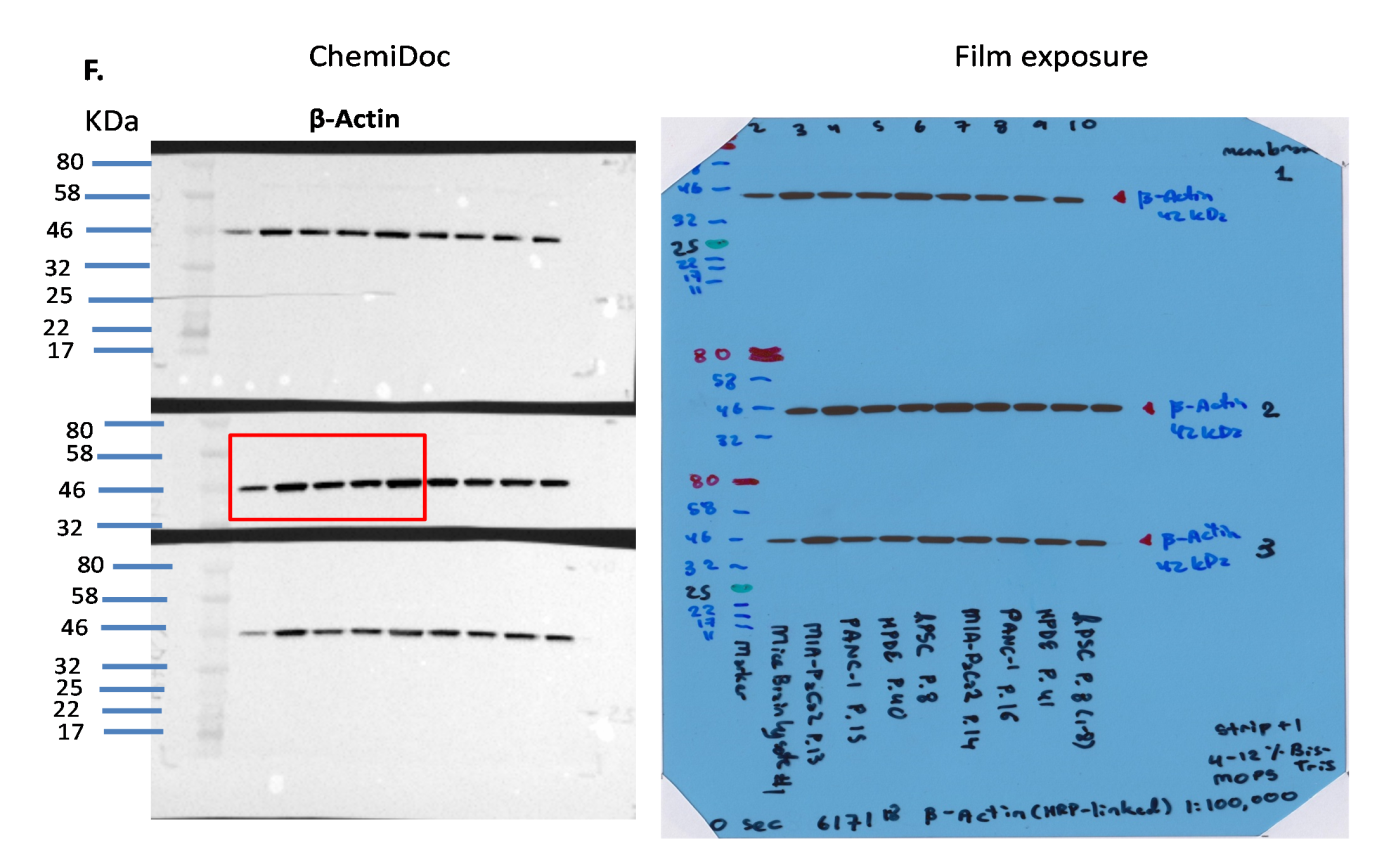


**Figure S7.** Full Western blot data presented in Figure 2A showing **(A)** Pan PMCA, **(B)** PMCA1, **(C-D)** PMCA2 and PMCA3, **(E)** PMCA4 and **(F)** β-actin. Western blot membranes were firstly exposed using ChemiDoc then followed by film exposure. The red box represents the Western blot area shown in Figure 2A. Protein band density was quantified using Imaged Lab software. Data showing raw band intensity and normalization to β-Actin are available in Supplementary Material 2.

**
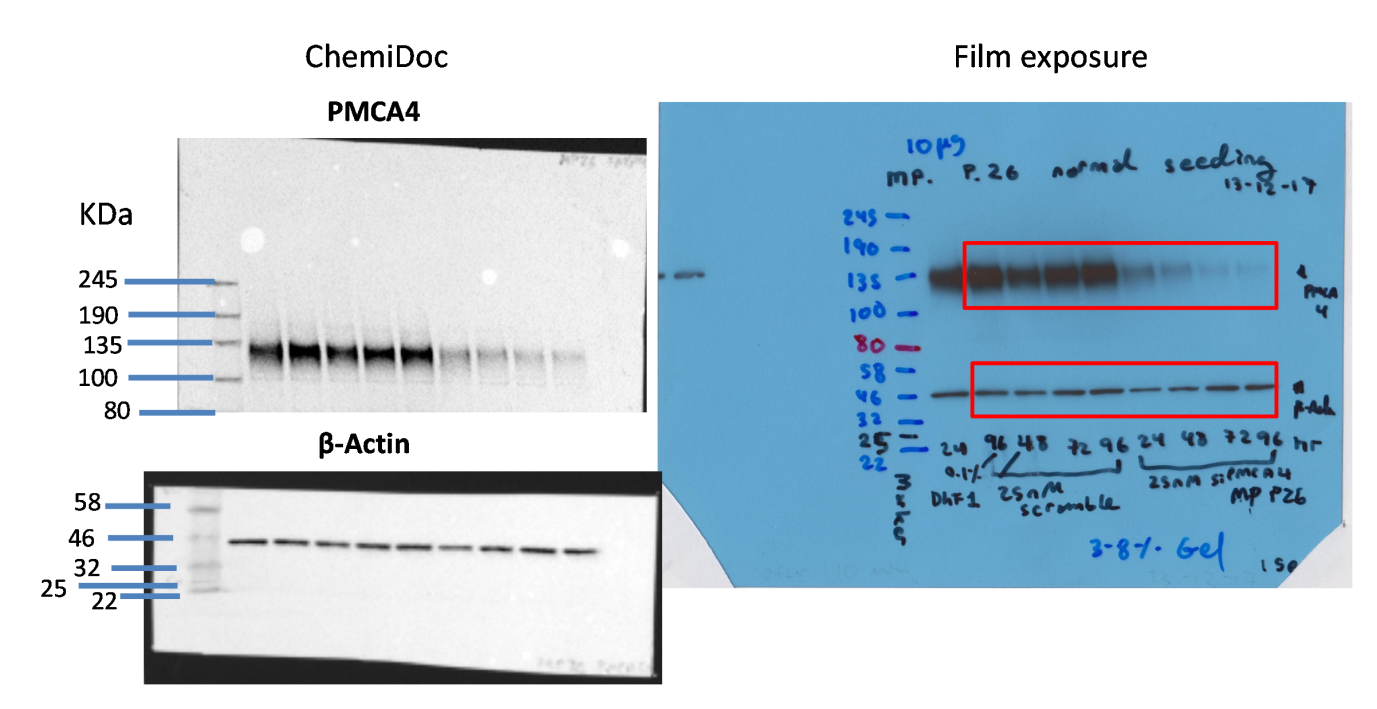
**

**Figure S8.** Full Western blot data presented in Figure 3A. Western blot membranes were firstly exposed using ChemiDoc then followed by film exposure. The red box represents the Western blot area shown in Figure 3A. Protein band density was quantified using Imaged Lab software. Data showing raw band intensity, normalization to β-Actin and comparison of PMCA4 expression between siPMCA4 and siNT (scramble control) are available in Supplementary Material 2.

**Supplementary References**

1. Mankad P, James A, Siriwardena AK, Elliott AC, Bruce JIE. Insulin protects pancreatic acinar cells from cytosolic calcium overload and inhibition of plasma membrane calcium pump. J Biol Chem. 2012 Jan 13;287(3):1823–36.

2. Barr R, Troxel KS, Crane FL. EGTA, a calcium chelator, inhibits electron transport in photosystem II of spinach chloroplasts at two different sites. Biochem Biophys Res Commun. 1980 Jan;92(1):206–12.

3. Moncoq K, Trieber CA, Young HS. The molecular basis for cyclopiazonic acid inhibition of the sarcoplasmic reticulum calcium pump. J Biol Chem. 2007 Mar 30;282(13):9748–57.

4. James AD. Metabolic regulation of the plasma membrane calcium pump in pancreatic ductal adenocarcinoma. University of Manchester; 2015.

5. Bruce JIE, Elliott AC. Oxidant-impaired intracellular Ca 2+ signaling in pancreatic acinar cells: role of the plasma membrane Ca 2+ -ATPase . Am J Physiol Physiol. 2007;293(3):C938–50.

6. Grynkiewicz G, Poenie M, Tsien RY. A new generation of Ca2+ indicators with greatly improved fluorescence properties. J Biol Chem. 1985 Mar 25;260(6):3440–50.

7. Huang Y, Putney JW. Relationship between intracellular calcium store depletion and calcium release-activated calcium current in a mast cell line (RBL-1). J Biol Chem. 1998 Jul 31;273(31):19554–9.

8. Tran NN, Leroy P, Bellucci L, Robert A, Nicolas A, Atkinson J, et al. Intracellular concentrations of fura-2 and fura-2/am in vascular smooth muscle cells following perfusion loading of fura-2/am in arterial segments. Cell Calcium. 1995 Nov;18(5):420–8.

9. Johnson I, Spence M (eds. . Indicators for Ca2+, Mg2+, Zn2+ and Other Metal Ions [Internet]. 11th ed. Johnson I, Spence MTZ, editors. The Molecular Probes ® Handbook. Life Technologies; 2010.

10. Agilent Technologies. Agilent Seahorse XF Real-Time ATP Rate Assay Kit [Internet]. 2018.

11. Agilent Technologies. Agilent Seahorse XF Real-Time ATP Rate Assay Report Generator User Guide [Internet]. 2018.

12. Agilent Technologies. Agilent Seahorse XF CO2 Contribution Factor Protocol User Guide [Internet]. 2017.

© 2019 by the authors. Licensee MDPI, Basel, Switzerland. This article is an open access article distributed under the terms and conditions of the Creative Commons Attribution (CC BY) license (http://creativecommons.org/licenses/by/4.0/).
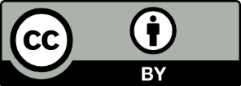

Supplement: Supplementary file 1 [file cancers-12-00218-s001.zip › cancers-654808-supplementary- CLEAN corrected.docx]
